# The positive effect of mother-performed infant massage on infantile eczema and maternal mental state: A randomized controlled trial

**DOI:** 10.3389/fpubh.2022.1068043

**Published:** 2023-01-11

**Authors:** Lin Lin, Lin Yu, Shuying Zhang, Jing Liu, Ying Xiong

**Affiliations:** ^1^Acupuncture and Massage College, Nanjing University of Chinese Medicine, Nanjing, Jiangsu, China; ^2^Pediatric Massage Department, Jiangsu Provincial Hospital of Chinese and Western Medicine, Nanjing, Jiangsu, China

**Keywords:** family massage, infant eczema, postpartum, anxiety, depression

## Abstract

**Objective:**

To observe the influence of MPIM on infantile eczema, quality of life, growth and maternal mental state.

**Methods:**

This trial was a randomized controlled study. Sixty-six full-term infants with eczema were randomly divided into eczema control group (EC group, *n* = 33) and eczema with MPIM group (EM group, *n* = 33), along with healthy full-term infants in the healthy control group (HC group, *n* = 31). The mothers in the EC group received the instruction of routine care, while the mothers in the EM group applied massage on the infants plus receiving the same instruction of the routine care. HC group received none of any specific intervention. Data were collected in the three groups at the baseline and at the end of 2- and 5-month intervention. Before and at the end of 2-month intervention, the following indexes were investigated in infants including the growth indexes, eczema area severity index (EASI), infantile dermatitis quality of life index (IDQOL). And the scores of self-rating anxiety scale (SAS) and self-rating depression scale (SDS) were investigated in mothers at the same timepoints. At the end of 5-month intervention, the infants' growth and relapse condition of eczema were observed.

**Results:**

Overall, 31 cases in HC group, 31 in EC group and 32 in EM group were included for data analysis. There were no significant differences in the indexes of infantile growth among the three groups (all *P* >0.05). The scores of EASI and IDQOL significantly lowered (both *P* < 0.001) in EC group following the instruction of routine care, along with reduced maternal scores of SAS and SDS (both *P* < 0.05). Compared with the EC group, the EM group showed significantly lower scores of EASI and IDQOL (both *P* < 0.001) and lower relapse rate (*P* < 0.01) in infants with eczema, along with significantly lower scores of SAS and SDS in mothers (both *P* < 0.01). Moreover, none of obvious adverse reaction was reported following MPIM, to which most of the mothers could adhere.

**Conclusion:**

MPIM could effectively promote the remission of infantile eczema and reduce its relapse, along with relieving maternal anxiety and depression mood.

**Clinical trial registration:**

Identifier: ChiCTR2200066246.

## Introduction

Eczema, also known as atopic eczema or atopic dermatitis, is a chronic relapsing inflammatory dermatosis characterized by pruritus, xerosis and a close association with immunoglobulin E (IgE)- mediated sensitization to aeroallergens and foods (1). The incidence of eczema in children has reached 15–20% (2), with the highest incidence in infants between the ages of 3 and 6 months old (2). A substantial portion of cases with eczema can go into complete remission by 2 years of age, while there are about 40% of cases with a prolonged duration and the highest risk for the atopic march (3). Moreover, infantile eczema obviously impairs infantile quality of life and potentially influence growth (4, 5). Current treatments for eczema aim to relieve symptoms since there is no cure for it (6, 7). General measures include the application of emollients and topical agents and avoidance of infections and trigger factors (2).

It is noteworthy that eczema in infants also negatively influences maternal mood (4). Postpartum mothers are especially susceptible to depressive and anxious episodes (8). It is reported that 8.5% of postpartum mothers experienced anxiety disorder (9) and 13% or higher experienced depression in their first postpartum year (10–12). Moreover, the mothers of infants with eczema are more susceptible to the psychological distress such as frustration, depression and anxiety, the level of which is often correlated to the eczema severity (4).

Infant massage, as a common traditional practice, is widely used all over the world for both preterm and full-term infants nowadays (13, 14). A series of studies have revealed its benefits to infants, such as enhancing growth and development, improving sleep and increasing interactions with parents (15–17). Previous trials also demonstrated that massage could relieve eczema symptoms in young children (18, 19). In China, infant massage is often applied on certain meridians and acupoints based on the theory of traditional Chinese medicine (TCM), which effectively relieves eczema in infants and toddlers (20–22). As we all know, infant massage can be performed not only by professionals in clinical setting, but also by parents at home (23). Systematic reviews recommend that parents can perform massage on low-risk infants for promoting mental and physical health due to its cost-effectiveness and no evidence of any risk (13, 14). Moreover, a series of studies indicated that mother-performed infant massage (MPIM) improved maternal depression, stress or negative mood during postpartum period (24–26). However, few trials have simultaneously observed the outcomes of both mothers and infants following MPIM. So far, there is also no trial to investigate whether MPIM can improve infantile eczema and whether MPIM influences the growth of infants with eczema.

Therefore, this trial was designed to observe the potential influence of MPIM on infantile eczema, growth and maternal mental state, which may be a beneficial health-care method for mother-infant dyads.

## Methods

### Trial design

**Figure 1:**
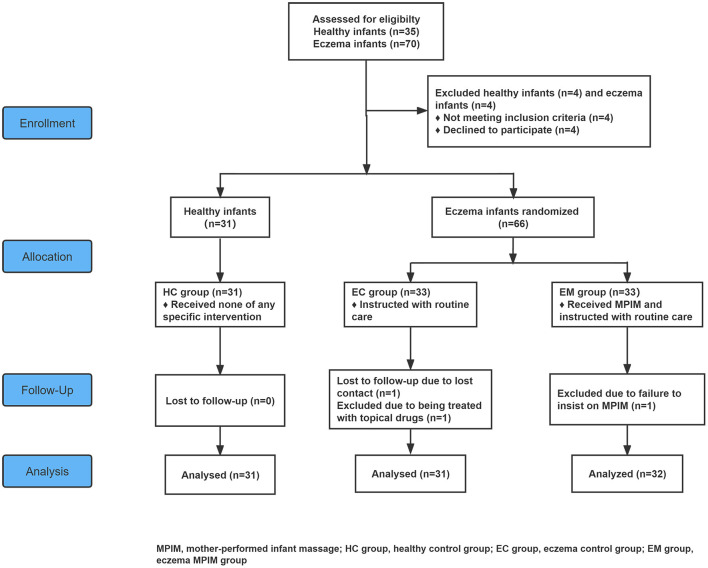
Flowchart of this study. MPIM, mother-performed infant massage; HC group, healthy control group; EC group, eczema control group; EM group, eczema MPIM group.

The study used a prospective block-controlled randomized design shown in Figure 1. Given convenient and practical implement, participants were recruited through publicity in Dingshan street community (Nanjing in Jiangsu province, China). This study was conducted in the health service center of Dingshan street community and at home, respectively based on the intervention protocol between April 2020 and March 2021. The optimal sample size of 87 (29 per group) was calculated by using PASS software (version 15.0, NCSS, USA) based on the following assumptions: β= 0.10, α= 0.05, σ1 = 1.8, σ2 = 0.4, μ1 = 1.6, and μ2 = 0.3, with 90% power, a two-sided alpha of 5%, and an estimated 20% loss to follow-up (27). Thirty-one healthy full-term infants and 66 full-term infants with acute eczema were enrolled in this study. Thirty-one healthy full-term infants were enrolled in the healthy control group (HC group, *n* = 31) for the comparison of infants' growth and mothers' mental state with eczema groups. By using a random number list produced by Excel software (version 2016, Microsoft, US), 66 infants with eczema were randomly divided into eczema control group (EC group, *n* = 33) and eczema with MPIM group (EM group, *n* = 33) in a 1:1 ratio by one investigator, who didn't participate in the following intervention or assessment. Participants were not blinded due to the nature of the intervention. The investigators responsible for the assessment of the infantile growth and eczema severity were blinded. However, the investigators responsible for the instruction of routine care and MPIM as well as regular supervision were not blinded due to the nature of the intervention.

### Inclusion criteria

For the infants with eczema:(1) full-term infant at or under 12 months old; (2) diagnosed as infantile eczema based on the clinical criteria for pediatric atopic dermatitis (28); (3) with informed consent and voluntary compliance with the study arrangement from the infant's mother.

For the healthy infants: (1) same as (1) and (3) for the infants with eczema above; (2) infants without any history of obvious visceral and functional diseases; (3) infants without eczema and other atopic diseases.

### Exclusion criteria

For the infants with eczema: (1) preterm infants or infant over 1 year old; (2) infant with any visceral disease or dysfunction except for eczema; (3) infant with infection, or the history of topical and systemic application of corticosteroids, antihistamines, antibiotics agents, traditional Chinese herbs or other specific intervention during the last 2 weeks; (4) infants with severe eczema, i.e., eczema area and severity index (EASI) score > 21 (2); (5) infant with obvious eczema or skin lesion on the back where massage is applied; (6) mother with diagnosed severe mental disorders or the scores of Zung's self-rating anxiety scale (SAS) and self-rating depression scale (SDS)≥70; (7) mother unable to follow study protocol.

For the healthy infants: same as (1), (3), (5), (6) and (7) for the infants with eczema above.

### Interventions

**Figure 2:**
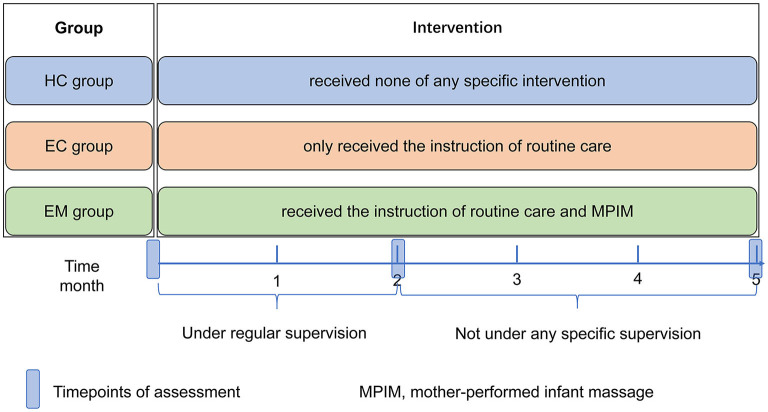
The protocol of the study.tif.

This study was a 5-month-long intervention study, which included the first 2-month intervention with regular supervision and the latter 3-month intervention without any supervision (shown in Figure 2). This kind of design aimed to have a knowledge of the feasibility and maternal adherence of MPIM, which is very important for promoting its application as a home-based healthcare method in the community. Data in the three groups were collected at the baseline and at the end of 2- and 5-month intervention to evaluate and analyze the outcome difference among groups. One investigator communicated with the mothers in the EC and EM group to enhance the adherence to the respective instruction by wechat communication once a week and by face to face once a month during the first 2-month intervention. During the latter 3-month intervention, the mothers in the EC and EM group were advised to record the adherence of respective intervention without any contact from investigators until the last assessment.

### Protocol in the HC group

Infants and mothers in the HC group received none of any specific intervention, except for the assessment at the three timepoints.

### Protocol in the EC group

The mothers in the EC group only received the instruction of routine care from one investigator for about half an hour in a small group of 3–5 mothers according to their convenience after the baseline observation. The tips of routine care for infantile eczema included the avoidance of skin irritants and allergens, avoidance of allergenic food in maternal and infantile diet, breast-feeding as much as possible and the application of emollient. For avoiding the confounder caused by using different emollients in this study, Pigeon skin lotion (Pigeon, Shanghai, CNH) was recommended in this study since it is popularly used on infants in China. It was advised to apply the skin lotion on the whole body of the infant except for the scalp at least twice daily, once during day time and once either after bathing or before sleep (if no bathing that day) in the evening. The used amount of the skin lotion was not fixed but determined by the body surface area and dry condition of skin in individual infant, which aimed to achieve the lubricant effect. In addition, let the infant lie on his/her stomach safely and comfortably for 10 min after the application of skin lotion before bedtime, for controlling the massaging position and time in EM group. The mothers in the EC group were instructed about infant massage by investigators after this study observation ended based on the voluntary rule.

### Protocol in the EM group

The mothers in the EM group were also instructed with the same routine care as the EC group and infant massage. The mothers were instructed with infant massage by the investigator for about 1 h in a small group of 3–5 mothers according to their convenience after the baseline observation, following the instruction about the same routine care as the EC group. The whole procedure of massage was determined according to the clinical application and textbook (29–32). The whole procedure took about 10 min as follows: Let the infant lie safely and comfortably on his/her stomach; the mother washed her hands and applied some skin lotion (Pigeon, Shanghai, CNH) to lubricate her hands and infant's back; firstly, the mother stroked the infant's back longitudinally along the middle line with finger bellies from shoulder level down to the sacrum level for 10 times; secondly, horizontally rubbed the back swiftly and gently with a palm for 20 times respectively at upper, middle, lower back levels; thirdly, applied traditional back-pinching manipulation (BP) for 6 repeats with the finger bellies; lastly, kneaded top-down on both sides of the back slowly and gently with a palm for 5 repeats. The details of one-repeat BP were as follows: pinched the skin located on the middle sacrum with the finger bellies and lifted to twist and move forward swiftly upward to the should level. The intensity of pinching and twisting would be increased gradually to avoid intolerable discomfort. For infants beyond 6 months, BP was performed for 9 repeats. After the instruction and learning, the mothers performed the whole procedure of infant massage on their infants for about 10 min in the presence of the investigator at the health care center. Afterwards, the mothers performed infant massage once daily at home 6 times per week. It was recommended to massage infants before bedtime in the evening as previous reports due to its beneficial effect on infantile sleep pattern (25). The investigator also communicated with the mothers on massage practice during wechat communication once a week and by face to face once a month. The mothers were also provided with massage video.

### Outcome observation

#### Weight, length, head circumference and BMI in infants

The weight, length and head circumference in infants were measured to assess infant's physical growth at the baseline, at the end of the 2- and 5-month intervention by one investigator, who was blinded from group division. The weight and length were measured by using an intelligent physical examination instrument (WS-RTG-ID, Wuhan Computer Software Development Co. LTD, China). Body mass index (BMI) was also calculated by using the following formula: BMI= weight (kg) /length (m)^2^. Head circumference was measured by a measuring tape (Guoshi measuring tape Co., LTD, China).

#### Eczema severity

Eczema severity was assessed by using the eczema area and severity index (EASI) at the baseline and the end of the 2-month intervention, which was conducted by one blinded investigator. EASI is a simple, reliable and easily understood system, which can be used by practitioners and investigators as a baseline evaluation and to track changes of eczema condition over time (2, 33, 34). EASI assesses the key signs of eczema including redness, thickness, excoriation, lichenification and the percentage of skin involving four areas (the head and neck, the trunk, the upper and lower extremities) (2, 33, 34). The scores are summed to achieve a total score ranging from 0 to 72. An EASI score ≤7 is considered as mild, 8–21 moderate, 22–50 severe, 51–72 very severe (2).

#### Quality of life in infants with eczema

The quality of life (QOL) in infants with eczema was evaluated by their mothers using the infants' dermatitis quality of life index (IDQOL) at the baseline and the end of the 2-month intervention. IDQOL is an easy and sensitive method with good reproducibility for parents to assess QOL impairment in infants with eczema (35). IDQOL contains 11 questions about current dermatitis severity, symptoms such as itching and scratching, mood, sleep, play, family activities, mealtimes, treatments, dressing and bathing. Ten questions present with the scores ranging from 0 to 3 and 1 question from 0 to 4 (35). In addition, the sleep condition in infants was also evaluated by the total scores of 2 questions involving sleep in IDQOL.

#### Mental state in mothers

Maternal anxiety and depression levels were evaluated, respectively by using SAS and SDS to investigate their mental state. Mothers in the three groups completed SAS and SDS questionnaires at the baseline and the end of the 2-month intervention. SAS and SDS were designed to quantify the severity of anxiety and depression symptoms, which is widely used as a common and effective self-assessment method (36–39). SDS is often used for perinatal women in China (40, 41). Both of SAS and SDS are 20-item self-reported assessment scales. The total score multiplied by 1.25 provides a standard score. The severity of symptoms was determined based on the standard scores of SAS and SDS as follows: < 50 (normal), 50–59 (mild), 60–69 (moderate), and ≥70 (severe). The score below 50 also indicates the levels of depression and anxiety mood (42, 43).

#### Adverse event

During the study, the mothers and investigators observed whether there were any adverse events in the infants after intervention, including any local impairment of skin, continuous crying, any abnormal change in sleep, eating and bowel movement.

#### Observation on the relapse of eczema

Since infantile eczema is a chronic relapsing inflammatory dermatosis (1), this study also observed the relapse condition of eczema in infants at the end of the 5-month intervention comparing with eczema condition at the end of the 2-month intervention. Persistent zero score of EASI from the end of 2-month to 5-month interventions was considered as complete remission. Eczema recurred after complete remission at the end of 2-month intervention was considered as a relapse. Persistent eczema without complete remission from the end of 2-month to 5-month interventions was considered as non-complete-remission. The general condition of infantile eczema was evaluated by a blinded investigator.

#### Statistical analysis

The one-way ANOVA and χ2 test were performed to compare baseline data among groups. The repeated-measures ANOVA was performed to compare infantile growth data among groups. The student's *t*-test was used to compare the scores of EASI, IDQOL and sleep in infant-mother dyads pre- and post-intervention among groups. The rank sum test was used to analyze the infantile eczema condition and the level of maternal anxiety and depression among groups. The SAS score and SDS score among groups were analyzed by one-way ANOVA and *t*-test for the comparison of pre- and post-intervention. Finally, we used spearman's rank correlation to assess the correlation between EASI score and the scores of IDQOL, sleep, SAS and SDS. All analyses were done by using Statistical Package for Social Sciences version 25.0 (SPSS, IBM, Armonk, NY, USA) and figures by using GraphPad Prism 6 (GraphPad Software Inc., La, Jolla, CA, USA). *P* < 0.05 were considered as statistical significance.

## Results

### Demographical data of infant-mother dyads

Thirty-one dyads and 63 dyads completed the study. The demographical profile of these infant-mother dyads is shown in Table 1. The age and the gender ratio in infants were not significantly different among the three groups (*F* = 0.464, *P* = 0.630; χ^2^ = 0.663, *P* = 0.718). There were no significant differences in the age, education background, primiparity and breast-feeding condition among the mothers in the three groups (*F* = 2.637, *P* = 0.077; χ^2^ = 1.274, 0.141 and 1.005, *P* = 0.693, 0.932 and 1.000).

**Table 1:** Demographical data of infant-mother dyads.

	**HC group (*n* = 31)**	**EC group (*n* = 31)**	**EM group (*n* = 32)**	***P*-value**
Infant				
Age (month)	4.3 ± 3.1	3.7 ± 2.9	3.6 ± 2.7	0.630
Male/female	14/17	17/14	17/15	0.718
Mother				
Age (year)	28.8 ± 2.6	27.8 ± 2.8	29.6 ± 3.6	0.077
Education background (high school and above/below)	30/1	28/3	29/3	0.693
Primiparity/multiparity	22/9	21/10	23/9	0.932
Breast-/mixed-/artificial feeding	27/2/2	27/3/1	28/2/2	1.000

Data are presented as the mean ± standard deviation or n/n.

HC, healthy control; EC, eczema control; EM, eczema with mother-performed infant massage.

### Weight, length, head circumference and BMI in infants

**Figure 3:**
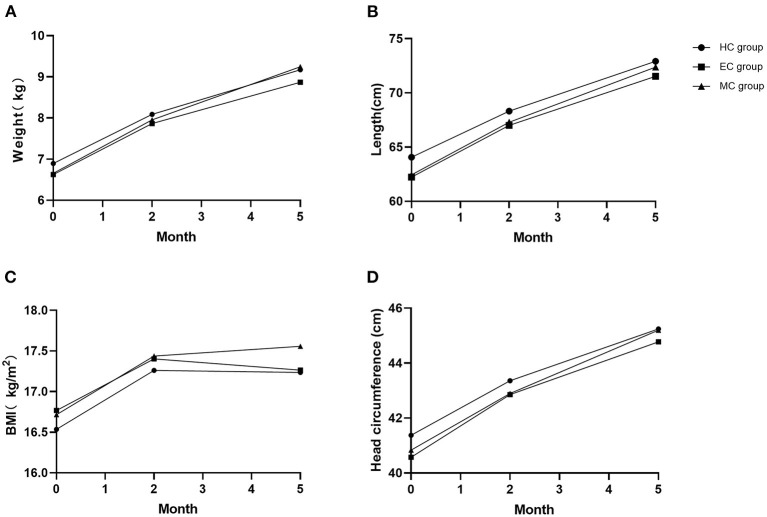
Comparison of infantile growth indexes at three timepoints. **(A)** Weight; **(B)** Length; **(C)** BMI; **(D)** Head circumference. No significant difference in all indexes among the three groups (P_A_ = 0.172, P_B_ = 0.381, P_C_ = 0.699, P_D_ = 0.370).

As shown in Figures 3A–D, there were no significant differences in the baseline values of infantile weight, length, BMI and head circumference in the three groups (*F* = 0.222, 0.768, 0.246 and 0.612; *P* = 0.802, 0.467, 0.782, and 0.544). The increasing in the infantile weight, length and head circumference was not significantly different among the three groups during this study (*F* = 1.752, 1.010 and 1.030; *P* = 0.172, 0.381 and 0.370). Moreover, the change in the infantile BMI value was not significantly different among the three groups (*F* = 0.490, *P* = 0.699).

### Improvement on infantile eczema

**Figure 4:**
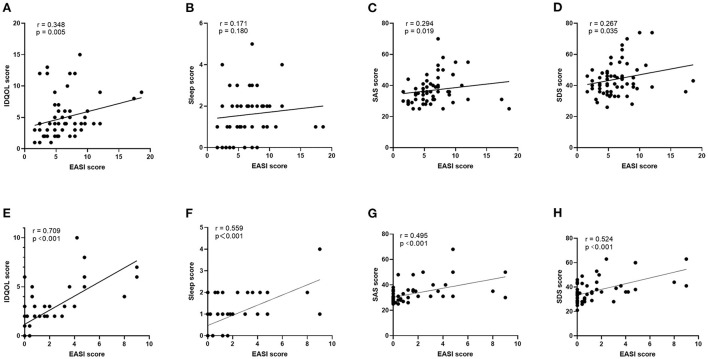
Correlation between EASI score in infant and indexes in infant-mother dyads at baseline and after 2-month intervention. Baseline/ After 2-month intervention: **(A,E)** EASI score and IDQOL score in infants; **(B,F)** EASI score and sleep score in infants; **(C,G)** EASI score and SAS score in mothers; **(D,H)** EASI score and SDS score in mothers.

The severity of infantile eczema was evaluated by EASI and IDQOL scores as shown in Table 2. At the baseline, the scores of EASI and IDQOL in infants were not significantly differenct between the EC and EM groups (t = 1.389, *P* = 0.172; t= 0.349, *P* =0.728). There were significantly lower scores of EASI (t = 8.749 and 15.460; both *P* < 0.001) and IDQOL (t = 5.981 and 8.132; both *P* < 0.001) after the 2-month intervention in both groups. Moreover, the EM group had significantly lower scores of EASI and IDQOL than the EC group (t = 3.953 and 3.797; both *P* < 0.001). Meanwhile, as shown in Table 3, the EM group had significantly lower scores involving sleep state following MPIM than the EC group (t = 3.969, *P* < 0.001). In addition, there was a significantly positive correlation of EASI scores with IDQOL scores before (γ = 0.348, *P* = 0.005, Figure 4A) and after 2-month intervention (γ= 0.709, *P* < 0.001, Figure 4E). There was also a significant correlation of EASI scores with sleep scores after 2-month intervention (γ= 0.559, *P* < 0.001, Figure 4F), but not at the baseline (γ= 0.171, *P* =0.180, Figure 4B). At the end of 5-month intervention, the EM group had significantly more cases of complete remission and fewer relapse cases than the EC group (*Z* = 3.124, *P* = 0.002, Table 4).

**Table 2:** Comparison of infantile eczema condition.

	**EC group (*n* = 31)**	**EM group (*n* = 32)**	***P*-value**
EASI score			
Baseline	7.0 ± 4.2	5.9 ± 1.9	0.172
2 Month	2.2 ± 2.7	0.3 ± 0.7	<0.001^***^
*P*-value	<0.001^***^	<0.001^***^	/
IDQOL score			
Baseline	4.8 ± 3.4	5.1 ± 3.1	0.728
2 Month	3.0 ± 2.4	1.1 ± 1.4	<0.001^***^
*P*-value	<0.001^***^	<0.001^***^	/

Data are presented as the mean ± standard deviation.

EASI, eczema area severity index; IDQOL, infantile dermatitis quality of life index.

*** *P* < 0.001.

**Table 3:** Comparison of sleep scores in infant-mother dyads.

	**EC group (*n* = 31)**	**EM group (*n* = 32)**	***P*-value**
Infant			
Baseline	1.42 ± 1.12	1.75 ± 1.02	0.224
2 Month	1.16 ± 0.90	0.38 ± 0.66	<0.001^***^
*P*-value	0.030^*^	<0.001^***^	/
Mother			
Baseline	5.61 ± 2.28	5.56 ± 1.98	0.919
2 Month	4.90 ± 1.76	4.03 ± 0.90	0.018^*^
*P*-value	0.039^*^	<0.001^***^	/

Data are presented as the mean ± standard deviation.

* *P* < 0.05,

*** *P* < 0.001.

**Table 4:** Comparison of infantile eczema condition at the end of 5-month intervention.

**Group**	**Complete remission**	**Relapse**	**Non-complete-remission**	***P*-value**
EC group (*n* = 31)	6 (19.35)	10 (32.26)	15 (48.39)	0.002^**^
EM group (*n* = 32)	15 (46.88)	13 (40.63)	4 (12.5)	

Data are presented as n (%).

** *P* < 0.01.

### Improvement on mental state in mothers

The mental state in mothers was evaluated by SAS and SDS scores as shown in Table 5. Compared with the HC group, the mothers in the EC and EM groups showed significantly higher scores of SDS (*P* = 0.032, *P* = 0.010) and non-significantly higher scores of SAS (*P* = 0.158, *P* = 0.236). At the end of 2-month intervention, there were significantly lower scores of SAS and SDS in the EC group (t = 2.087, *P* = 0.046; t = 2.695, *P* = 0.011) and EM group (t = 6.066 and 7.972, both *P* < 0.001). The EM group had significantly lower scores of SAS and SDS than the EC group (*P* = 0.003, *P* = 0.002). Intriguingly, the scores of SAS and SDS in EM group were significantly lower at the end of 2-month intervention than those in the HC group (*P* = 0.031, *P* = 0.039). The scores of SAS and SDS in the HC group didn't show significant change (t = 0.220, *P* = 0.827; t = 0.000 *P* = 1.000). Moreover, compared with the EC group, the ratio of cases with mild-to-moderate depression to normal cases lowered significantly (*Z* = 2.349, *P* = 0.019), but the ratio of cases with mild-to-moderate anxiety to normal cases didn't change significantly (*Z* = 1.016, *P* = 0.310, Table 6). Meanwhile, the correlation of maternal scores of SAS and SDS with infantile EASI scores showed significantly positive before (γ= 0.294, *P* < 0.019; γ= 0.267, *P* < 0.035; Figures 4C,D) and after 2-month intervention (γ= 0.495, *P* < 0.001; γ= 0.524, *P* < 0.001, Figures 4G,H). In addition, as shown in Table 3, maternal sleep state got improved remarkably in the EC and EM groups after 2-month intervention (t = 2.160, *P* = 0.039; t = 4.923, *P* < 0.001,). MPIM further improved maternal sleep state (t = 2.468, *P* = 0.018).

**Table 5:** Comparison of SAS and SDS scores.

	**HC group (*n* = 31)**	**EC group (*n* = 31)**	**EM group (*n* = 32)**	***P*-value**	***P*-value^a^**	***P*-value^b^**	***P*-value^c^**
SAS score							
Baseline	34.1 ± 8.3	37.3 ± 9.6	36.7 ± 8.6	0.316	0.158	0.236	0.809
2 Month	33.7 ± 8.2	35.4 ± 9.3	29.5 ± 4.9	0.009^**^	0.390	0.031^*^	0.003^**^
*P*-value	0.827	0.046^*^	<0.001^***^	/	/	/	/
SDS score							
Baseline	37.7 ± 10.7	43.6 ± 2.1	44.8 ± 9.7	0.023	0.032	0.010	0.663
2 Month	37.7 ± 10.8	40.3 ± 10.2	32.9 ± 6.2	0.007^**^	0.275	0.039^*^	0.002^***^
*P*-value	1.000	0.011^*^	<0.001^***^	/	/	/	/

Data are presented as the mean ± standard deviation.

SAS, self-rating anxiety scale; SDS, self-rating depression scale.

^a, b and c^ represent P-values for comparisons between HC and EC group, HC and EM group, EC and EM group, respectively.

* *P* < 0.05,

** *P* < 0.01,

*** *P* < 0.001.

**Table 6:** Comparison of maternal depression and anxiety condition.

	**group**	**Severity**					
		**Normal**	**Mild-to-moderate**	***P*-value**	**Normal**	**Mild-to-moderate**	***P*-value**
		**Pre-intervention**			**Post-intervention**		
Anxiety	EC group (*n* = 31)	28 (90.32)	3 (9.68)	0.618	30 (96.77)	1 (3.23)	0.310
	EM group (*n* = 32)	30 (93.75)	2 (6.25)		32 (100)	0 (0)	
Depression	EC group (*n* = 31)	26 (83.87)	5 (16.13)	0.565	26 (83.87)	5 (16.13)	0.019^*^
	EM group (n=32)	25 (78.13)	7 (21.88)		32 (100)	0 (0)	

Data are presented as n (%).

* *P* < 0.05.

### Safety observation of MPIM

During the study, none of any obviously adverse events was observed and reported following MPIM or routine care except for temporal crying during BP only on the first several days in 4 cases in the EM group.

### Adherence in routine care or MPIM

This study was also designed to observe maternal adherence condition during the latter 3-month intervention without any supervision from investigators. The investigator asked about (1) whether the mothers persisted in following instructed routine care in the EC and EM groups; (2) how often MPIM was conducted in the EM group. It was found that routine care was followed by all mothers in both groups. Nineteen mothers performed MPIM at least 6 days a week, 12 mothers 1 or 2 days a week, and only 1 mother discontinued for no special reason.

## Discussion

This study aimed to observe the potentially positive effect of MPIM on infantile eczema, growth and the mental state in mothers. This study indicated that infantile eczema impaired infantile quality of life and negatively influenced maternal mental state. Infantile eczema improved over time after mothers followed the instructions about the routine care for infantile eczema, along with improved depressive and anxious mood in mothers. More importantly, this study demonstrated that MPIM further enhanced eczema remission and decreased its relapse rate, together with further improved mental state in mothers. However, the growth in infants with eczema was not affected by MPIM.

Eczema is typically the first allergic manifestation to appear (44). Precipitating or aggravating factors of eczema include food allergens, environmental allergens or irritants, climatic condition, stress and genetic predisposition, although the exact cause of eczema is not clear (45). Infantile emollient is inexpensive, widely available, and used extensively for relieving eczema (46), which can improve the function of skin barrier and reduce itch and irritation (45). About 35% to 40% of children with moderate to severe eczema have food allergy, and eczema can be improved significantly by eliminating the causative food from their diet (46). Previous studies demonstrated that breastfeeding was associated with lower incidences of allergic diseases, eczema included (47, 48). Breast-feeding is proved to prevent allergy due to the immune mediators and oligosaccharides in maternal milk, which facilitates balanced gut microbiota to induce tolerance (44). In this study, infantile eczema improved after the mothers followed these instructions about the routine care for infantile eczema. Itching, the cardinal symptom of eczema, obviously impairs infantile QOL, therefore, IDQOL is widely used in conjunction with EASI for assessing clinical severity of eczema (4, 5). This study also demonstrated that QOL of infants with eczema was impaired by eczema and improved along with relieved eczema after the mothers followed the instruction of routine care.

As we all know, postpartum mothers are susceptible to depression and anxiety episodes (9–12), whose mood is negatively influenced by infant eczema (4). This study showed that the respective prevalence of anxiety and depression among these postpartum mothers was 1/31 and 3/31 in HC group, 3/31 and 5/31 in EC group, 2/32 and 7/32 in EM group at baseline, which are consistent with previous reports (10–12). The mothers in the EC and EM groups had significantly higher levels of depression and anxiety mood than those in the HC group at baseline. Moreover, the mothers in the EC and EM groups had higher rates of depression symptoms than those in the HC group at baseline, but not higher rates for anxiety symptom. This result indicated that infantile eczema might increase the susceptibility of mothers to depression, which is consistent with previous study (4).

Two previous trials demonstrated that mother-performed massage could relieve eczema symptoms in young children (18, 19). One trial showed that the children with eczema were massaged with skin oil on the whole body (except for head and face) by the therapist once a week and by mothers every day for 8 weeks, which decreased night-time disturbance score (18). The other one demonstrated that mother-performed massage on the whole body (except for head) for 1 month after the first instruction by therapist could relieve eczema symptoms in children and reduce the anxiety of mothers and children. In China, infant massage is applied based on the theory of TCM, which is performed on the specific areas to relieve eczema in infants and toddlers (20–22). So far, there is no trial to investigate whether mother-performed massage can relieve infantile eczema. It is well-known that the development of eczema is associated with Th2-skewed inflammation, which is closely related with intestinal dysbiosis (49, 50). Our previous experiments demonstrated that BP, the major manipulation in MPIM, attenuated Th2-skewed inflammation and regulate the intestinal dysbiosis in immature rats with allergic airway inflammation (51, 52). In TCM, the back is the location where Du vessel and Bladder meridian run, which can be stimulated to regulate visceral function and relieve allergic symptoms (29, 32). Based on our previous trials and animal experiments, this study was designed to massage the back (29–31). This study demonstrated that MPIM further enhanced eczema remission and decreased its relapse rate, along with improved infantile QOL. It is worth mentioning that MPIM remarkedly improved the sleep state of infants with eczema and their mothers, which is consistent with previous studies (53–56). Our previously trial showed mother-performed massage improved the depression and anxiety state of the mothers of asthmatic children (31). This study indicated that MPIM also significantly reduced the levels of maternal depression and anxiety mood, including obvious depression symptom, which consist with previous trials (24–26, 57, 58).

Further investigation in this study revealed the positive correlation between the levels of depression and anxiety and the severity of infant eczema. Therefore, on one side, the sleep state of infants got better with eczema improvement, which might beneficially influence the mothers sleep and mood. On the other hand, skin-to-skin contact during MPIM might trigger oxytocin (OT) production and release, which contributed to anti-stress effect and improving sleep state in mothers as previous reports (59–61). OT is synthesized and released from the magnocellular neurons of the paraventricular (PVN) and supraoptic nuclei (SON) of the hypothalamus (62). OT has positive central effects on psychological adjustment and maternal behaviors during postpartum period (63–67). It is also believed that tactile contact between mother and child during massage could reduce the levels of stress-related hormones (cortisol and norepinephrine) in children and mothers and led to relieved eczema and maternal anxiety (19, 68). The placebo effect could also play a role due to a beneficial expectation (18).

Few trials investigated the outcomes of both infants and mothers following MPIM. To our knowledge, only one trial did investigate the physical status of preterm infants and the psychological state in the mothers following MPIM, which led to greater weight, motor development, and larger bicep and thigh circumference in infants as well as increased maternal attachment and decreased anxiety compared to the control group (69). However, our study showed that the growth of the infants with eczema was not affected by eczema during 5-month observation in this study. MPIM didn't significantly enhance the infantile growth although it improved infantile eczema. Previous reports also demonstrated inconsistent results about the effect of MPIM on enhancing infantile growth in preterm infants and healthy infants. Gonzalez (70) and Zhang (71) reported that MPIM could enhance the growth of preterm infants while Abedi (72) reported that MPIM didn't enhance the growth of healthy neonates. In this study, most of infants had mild eczema and thus their growth might not be affected by eczema, which might explain the result.

In addition, the correlations of EASI score with infantile scores of IDQOL and sleep and maternal scores of SAS and SDS were more significant after 2-month intervention compared with those at baseline. This result suggests that infantile quality of life and sleep and maternal mood might be affected easily by surrounding complicated factors during early postpartum stage, which may change overtime.

Previous study showed that parents preferred to learn and practice infant massage on their own babies either in a class, in a hospital or at home under the investigators' supervision and instruction (24–26, 30, 31, 73). However, for the feasibility and practicality, the adherence of MPIM at home without any specific supervision of investigators should be investigated, which is the minor aim of this study. This study demonstrated good adherence of MPIM without persistent supervision from investigators, which indicated that MPIM is feasible and convenient to implement at home after the relative training in the community background.

## Limitations

There are some limitations in this study. Firstly, for the convenient implementation in this pilot study, it was designed to enroll participants from one community, which might influence the real intervention effects. Secondly, this study was designed not to supervise the implementation of MPIM during the latter 3 months, aiming to observe the feasibility and adherence of MPIM. Therefore, the outcomes of infant-mother dyads at the end of 5-month intervention might be affected by the various performing frequency of MPIM. Thirdly, due to the small sample size, this study didn't analyze the potentially different effects caused by various frequency of MPIM at the end of 5-month intervention. Fourthly, this pilot study only enrolled infants and their mothers to observe the potential effect on maternal mental state. It can also extend to fathers of eczema infants who also experience worse mental state during postpartum. Fifthly, due to the study feature, mothers could not be blinded and they also assessed their own mental state and infantile quality of life, which might bring certain placebo effect. In the future, multi-centered randomized controlled trials with larger size are warranted to further investigate the potential benefits of parent-performed infant massage on the outcomes of infant-parent dyads for a prolonged time.

## Conclusion

In conclusion, this study demonstrated for the first time that MPIM enhanced the remission of infantile eczema, reduced the relapse rate and improved maternal depression and anxiety mood. Given its safety, cost-effectiveness and feasibility, MPIM may be recommended as a routine home healthcare method for infants with eczema in the community background.

## Data availability statement

The original contributions presented in the study are included in the article/supplementary material, further inquiries can be directed to the corresponding author.

## Ethics statement

The studies involving human participants were reviewed and approved by Ethics Committee of Jiangsu Provincial Hospital of Intergrated Chinese and Western Medicine. Number of ethics approval: 2020LWKY010. Written informed consent to participate in this study was provided by the participants' legal guardian/next of kin.

## Author contributions

YX contributed to the conception and design of the study. LY and JL participated in the design of the manuscript and collected data. LL and YX drafted the manuscript. LL and SZ analyzed data. All authors contributed to manuscript revision, read, and approved the submitted version.
